# Whole body MR imaging in ankylosing spondylitis: a descriptive pilot study in patients with suspected early and active confirmed ankylosing spondylitis

**DOI:** 10.1186/1471-2474-8-20

**Published:** 2007-02-27

**Authors:** Ulrich Weber, Christian WA Pfirrmann, Rudolf O Kissling, Juerg Hodler, Marco Zanetti

**Affiliations:** 1 Department of Rheumatology, Balgrist University Hospital, Forchstrasse 340, 8008 Zurich, Switzerland; 2 Department of Radiology, Balgrist University Hospital, Forchstrasse 340, 8008 Zurich, Switzerland

## Abstract

**Background:**

Ankylosing spondylitis is a chronic inflammatory rheumatic disorder which usually begins in early adulthood. The diagnosis is often delayed by many years. MR imaging has become the preferred imaging method for detection of early inflammation of the axial skeleton in ankylosing spondylitis.

The goal of this study was to assess the frequency and distribution of abnormalities on whole body MR imaging in patients with suspected early ankylosing spondylitis and with active confirmed ankylosing spondylitis.

**Methods:**

Ten patients with suspected early ankylosing spondylitis and ten patients with confirmed ankylosing spondylitis were enrolled. On an 18-channel MR system, coronal and sagittal T1 weighted and STIR sequences were acquired covering the entire spine, sacrum, anterior chest wall, shoulder girdle, and pelvis. The total examination time was 30 minutes.

**Results:**

In both groups inflammatory lesions of the lower thoracic spine were frequent (number of patients with suspected early/confirmed ankylosing spondylitis: 7/9). In confirmed ankylosing spondylitis the upper thoracic spine (3/6) and the lumbar spine (4/8) were more commonly involved. The inferior iliac quadrant of the sacroiliac joints was frequently altered in both groups (8/8). The superior iliac (2/5), inferior sacral (6/10) and superior sacral (3/6) quadrants were more frequently affected in confirmed ankylosing spondylitis. Abnormalities of the manubriosternal joint (2/4), the sternoclavicular joints (1/2) and hip joint effusion (4/3) were also seen.

**Conclusion:**

In both suspected early ankylosing spondylitis and confirmed ankylosing spondylitis, whole body MR examinations frequently demonstrate inflammatory lesions outside the sacroiliac joints. These lesions are similarly distributed but occur less frequently in suspected early compared to confirmed ankylosing spondylitis. Due to the small sample size in this pilot study these results need to be confirmed in larger studies with this emerging technique.

## Background

Ankylosing spondylitis (AS) is a chronic inflammatory rheumatic disorder mainly affecting the sacroiliac (SI) joints and the spine. The leading symptom is inflammatory back pain. The impairment of quality of life and the socioeconomic consequences may be substantial [1-4].

Recently, symptomatically highly effective drugs such as tumor necrosis factor alpha (TNFα) inhibitors have gained increasing interest for treatment of AS [5]. Imaging may play an important role in assessing disease activity at an early stage and for the follow-up of the disorder under these recently introduced treatment options. However, the definition of an early stage of AS represents a methodological problem because there are no validated classification or diagnostic criteria [6]. Inflammatory back pain (IBP) is the leading early symptom of the closely interrelated forms of spondyloarthropathies, AS being the most common entity of this disease group [7].

The diagnosis of AS is still delayed by more than seven years in many patients [8,9]. MR imaging has become the preferred imaging method for detection of early inflammation of the spine and the SI joints in AS [10-16]. The spinal segment most commonly affected by AS appears to be the thoracic spine [13,17-19].

Advances in multichannel technology allow to perform whole body MR imaging with visualization of inflammatory lesions of the entire spine, the shoulder girdle, the anterior chest wall and the pelvis, including the SI joints, symphysis pubis and hip joints in a single examination.

Whole body MR imaging may provide characteristic patterns of abnormalities in early and late AS in various regions outside the SI joint which may contribute to early diagnosis in patients with suspected AS. We hypothesized that patients with suspected early AS may demonstrate a similar pattern of abnormalities compared to patients with longstanding and confirmed AS. Thus, the aim of this study was to assess the frequency and patterns of abnormalities on whole body MR imaging in patients with suspected early AS and active confirmed AS.

## Methods

### Study population

Ten patients with suspected early AS and ten patients with active confirmed AS were prospectively recruited in our outpatient department of rheumatology between November 2004 and August 2005 from a national population-based prospective observational registry of AS patients (Swiss Clinical Quality Management in Ankylosing Spondylitis SCQM AS) which started in August 2004. Participants in this registry were enrolled by practicing or hospital based board certified rheumatologists.

The responsible institutional review boards (spezialisierte Unterkommission Orthopaedie/Bewegungsapparat der kantonalen Ethikkommission Zuerich; Ethik-Kommission [KEK], Gesundheitsdirektion Kanton Zuerich) approved the protocol, and all patients gave written informed consent.

### Inclusion criteria

Patients were enrolled in the suspected early AS group if they were suffering from inflammatory low back pain for more than three months and for less than two years and if at least one of the following features was present: Rapid and substantial pain relief by non-steroidal antiinflammatory drugs, alternating buttock pain, peripheral arthritis, enthesitis, dactylitis, uveitis, HLA B27 status positive, elevated acute phase reactants, family history of AS. Inflammatory back pain was defined as relapsing or persisting low back pain, improving with exercise but not relieved by rest, morning stiffness of at least 30 minutes and awakening in the second half of the night due to low back pain. At least two of the three latter criteria had to be fulfilled by the patients in the group with suspected early AS.

The term of suspected early AS was chosen because there are no validated criteria for the diagnosis of early AS. The commonly used modified New York classification criteria [20] for AS use radiographic evidence of sacroiliitis as the main item. Relapsing or persisting inflammatory back pain is considered to be an early symptom of the closely interrelated forms of spondyloarthropathies, AS being the most common entity in this disease group.

Patients with confirmed AS were enrolled if they fulfilled the modified New York criteria [20].

The minimal disease activity required for both groups to be enrolled was a Bath Ankylosing Spondylitis Disease Activity Index (BASDAI) of at least 4 and/or spine pain of at least 4 (BASDAI item 2: second question of the BASDAI instrument dealing with back pain) on a numeric rating scale ranging from 0 to 10 [21]. A BASDAI value of 4 or higher is considered to reflect a high disease activity.

### Exclusion criteria

Exclusion criteria were clinical signs of psoriasis, SAPHO syndrome (synovitis, acne, pustulosis, hyperostosis, osteitis), chronic inflammatory bowel disease (Crohn's disease, ulcerative colitis) or reactive arthritis; previous or ongoing treatment with tumor necrosis factor alpha (TNFα)-inhibitors or other biologics; previous surgery of the spine, pelvis or shoulder girdle; pregnancy within the first 3 months, contraindications for MR imaging including cardiac pacemakers and neurostimulators.

### MRI protocol

Whole body MR imaging was performed in all patients using coronal and sagittal T1 weighted and short tau inversion recovery (STIR) sequences of the entire spine and sacrum, anterior chest wall and shoulder and pelvis. MR imaging was performed on a 1.5 Tesla magnet (Siemens Medical Solutions, Erlangen, Germany), equipped with 18 independent radiofrequency channels. A body matrix coil was used. Coronal T1 weighted spin echo (TR: 571 ms, TE: 12 ms, PAT Factor 2, PAT mode GRAPPA [generalized autocalibrating partially parallel acquisition]) and coronal Turbo STIR (TR: 9860 ms, TE: 99 ms, TI: 130 ms, Turbo Factor 21, PAT Factor 2, PAT mode GRAPPA) sequences were acquired using two imaging steps with a FOV of 450 mm × 450 mm and an imaging Matrix of 348 × 267 pixels per step, 5 mm section thickness, interslice gap 1 mm, 31 sections resulting in a FOV of 785 × 450 mm. Sagittal T1 weighted spin echo (TR: 401 ms, TE: 11 ms, PAT Factor 2, PAT mode GRAPPA) and sagittal turbo STIR (TR: 6270 ms, TE: 93 ms, TI: 130 ms, Turbo Factor 21, PAT Factor 2, PAT mode GRAPPA) were acquired using two imaging steps with a FOV of 450 mm × 450 mm and an imaging matrix of 512 × 256 pixels per step, 4 mm slice thickness, interslice gap 0.4 mm, 20 sections resulting in a FOV of 792 × 450 mm. The sum of the acquisition times for all sequences including two localizers was 21 minutes 53 seconds. The total examination time including patient positioning was 30 minutes.

### Analysis of MR images

The MR images were analyzed by consensus between two staff musculoskeletal radiologists (CP, MZ), who were blinded with regard to the two patient groups. The films were read in random order at a work station. Assessments included inflammatory changes (edema-like signal abnormalities of bone marrow) of the spine and around the SI joints as well as fatty replacement of subchondral bone marrow of the SI joints. Assessment for the shoulders, anterior chest wall and pelvis included edema-like signal abnormalities of adjacent bone marrow and joint effusion.

Spinal assessment was based on 23 vertebral units from C2/C3 to L5/S1. A vertebral unit was defined as the region between two virtual lines drawn through the middle of each vertebra according to one of the recently published MR scoring systems for inflammatory involvement of the spine in AS [19]. Bone marrow signal abnormalities in both adjacent vertebral bodies of the vertebral unit were assessed irrespective of the extent of the lesions (possible range: 0 to 23 lesions per patient). Inflammatory changes of the spinous processes and the facet joints were noted.

The SI joints were divided into four quadrants each (superior and inferior iliac and sacral quadrants) regardless of the extent of the lesions (possible range: 0 to 8 lesions per patient).

In the anterior chest wall the sternoclavicular, costosternal and manubriosternal joints were assessed. In the shoulders the glenohumeral and acromioclavicular joints as well as the subacromial bursa were included in the evaluation. In the pelvis both hips and trochanteric regions and the symphysis pubis were evaluated. Abnormal hip effusion was defined as fluid adjacent to the entire length of the femoral neck, measuring at least 5 mm in width [22].

### Statistics

We compared means and medians. Since they did not fit well to a normal distribution, we used a non-parametric equality of medians test. It tests the null hypothesis that the two samples (suspected early AS and confirmed AS) were drawn from populations with the same median. A computer software package (SPSS, version 11.5.0; SPSS, Chicago, IL) was used to perform all statistical calculations.

## Results

### Patient characteristics (Table 1)

Ten patients had suspected early AS with a median disease duration of 0.8 years (range 0.3 to 2.0 years; seven male and three female patients; median age 28.5 years). Ten patients had confirmed AS with a median disease duration of 12.5 years (range 6 to 28 years; seven male and three female patients; median age 36.5 years).

The group with suspected early AS had a median BASDAI of 4.6 (median spinal pain 6.0 according to BASDAI 2, the second question of the BASDAI instrument dealing with back pain; median erythrocyte sedimentation rate 8.0 mm/h). The confirmed AS group had a median BASDAI of 5.9, a median BASDAI 2 of 7.0 and a median erythrocyte sedimentation rate of 16.5 mm/h.

The parameters of disease activity showed no statistically significant differences between the two groups. A significant difference in parameters of metrology (BASMI [Bath Ankylosing Spondylitis Metrology Index; [24]]) most probably reflects the longer disease duration of the confirmed AS group.

### MR findings of the spine (Table 2; Figure 1, 2, 3)

Inflammatory lesions of the lower thoracic spine were frequent in both groups: Fifteen lesions in 7 patients of the group with suspected early AS and 21 lesions in 9 patients of the confirmed AS group. Patients with confirmed AS had more inflammatory lesions of the upper thoracic spine (17 lesions in 6 patients versus 6 lesions in 3 patients in the suspected early AS group) and the lumbar spine (18 lesions in 8 patients versus 5 lesions in 4 patients in the suspected early AS group). Inflammatory involvement of the cervical spine was rare with 5 lesions in 3 patients of the confirmed AS group and 2 lesions in 2 patients of the suspected early AS group.

Inflammatory lesions of the spinous processes and the facet joints of the spine were more common in the confirmed AS group. Involvement of the spinous processes was found in 5 patients (9 lesions) in the confirmed AS group (no findings in the suspected early AS group) and involvement of the facet joints in 2 patients (4 lesions) in the confirmed AS group versus 1 patient (2 lesions) in the suspected early AS group.

### MR findings of the SI joints (Table 2)

SI joints showed frequent inflammatory lesions in the inferior iliac quadrant in both groups. There were 13 lesions in 8 patients in the suspected early AS group and 16 lesions in 8 patients in the confirmed AS group. The remaining three sacroiliac quadrants were more frequently affected in confirmed AS (confirmed AS group versus suspected early AS group). There were 8 lesions in 5 patients versus 2 lesions in 2 patients in the superior iliac quadrant; 15 lesions in 10 patients versus 8 lesions in 6 patients in the inferior sacral quadrant and 9 lesions in 6 patients versus 3 lesions in 3 patients in the superior sacral quadrant.

**Figure 4:**
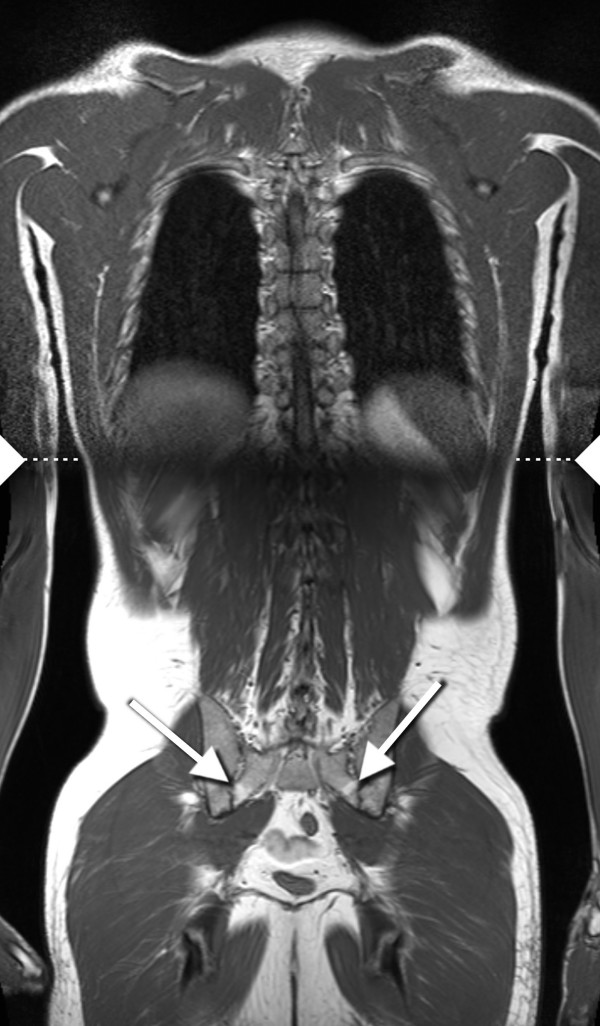
47-year old male patient with confirmed AS (duration of inflammatory back pain 13 years, BASDAI 7.0, HLA B27 positive). Fatty replacement of subchondral bone marrow is shown in the lower sacral quadrant (arrows) on the coronal T1-weighted image.

Fatty replacement of subchondral bone marrow of the SI joints (Figure 4) was more frequent in the confirmed AS group (confirmed AS group versus suspected early AS group). In the inferior iliac quadrant there were 10 lesions in 6 patients versus 4 lesions in 3 patients. The corresponding numbers were 2 lesions in 2 patients in both groups for the superior iliac quadrant, 17 lesions in 9 patients versus 5 lesions in 4 patients in the inferior sacral quadrant and 9 lesions in 6 patients versus 3 lesions in 3 patients in the superior sacral quadrant.

### MR findings of the anterior chest wall (Table 2; Figure 5)

Inflammatory changes of the anterior chest wall were found (suspected early AS group versus confirmed AS group) in the manubriosternal joint in 6 patients (2 versus 4), in the sternoclavicular joints in 3 patients (1 versus 2) and in the costosternal joints in 1 patient in the suspected early AS group.

### MR findings of the shoulder (Table 2)

Inflammatory changes of the shoulder were rare and confined to the confirmed AS group. Abnormalities of the acromioclavicular joint were seen in three patients and of the subacromial bursa in one patient. None of the glenohumeral joints was affected.

### MR findings of the pelvis (Table 2; Figure 6)

Seven patients had hip effusions (4 patients in the suspected early AS group and 3 patients in the confirmed AS group). In a single patient each with confirmed AS edema-like abnormalities of the left femoral head and acetabulum, the greater trochanter as well as the symphysis were found.

### MR findings suggestive of inflammatory lesions with regard to different localizations (Figure 7)

In both groups the highest percentage of patients with inflammatory lesions was observed using the single regions SI joints or thoracic spine. In this small sample size a combination of the regions SI joints and any spinal area detected all patients with inflammatory lesions.

## Discussion

The diagnosis of AS may be delayed by more than seven years [8,9]. The modified New York classification criteria [20] are often used for diagnostic purposes. They depend on the presence of sacroiliitis on standard radiographs which develops late in the course of the disease [25,26]. There is a need for new criteria for early diagnosis of AS [6].

The term of suspected early AS was chosen in this study because there are no validated criteria for the diagnosis of early AS. Relapsing or persisting inflammatory back pain is considered to be an early symptom of the closely interrelated forms of spondyloarthropathies, AS being the most common entity in this disease group [7]. It will take many years of follow-up of these 10 patients with suspected early AS participating in a prospective observational registry to confirm the diagnosis of AS or another form of spondyloarthritis. In a cohort of 88 patients with possible AS (inflammatory low back pain and at least one additional feature suggestive of spondyloarthritis), radiological sacroiliitis became evident only after a mean disease duration of nine years [26].

In recent years MR imaging has proved to be a major advance in detection of early inflammation of the spine and the SI joints in AS [10-16]. If signal abnormalities of bone marrow represent a specific inflammatory feature of AS is controversial [27]. A correlation between clinical disease activity and acute inflammatory changes of the SI joints and the spine has been found in some studies [19,28] contrasting with negative findings in others [18,29]. Additional data are needed if the apparently inflammatory lesions seen on MR imaging are indeed predictive of radiographic changes diagnosed some years later [14]. The quality of information gained by the use of STIR as the sole technique in imaging of inflammatory lesions of the spine in confirmed AS was not inferior to the use of gadolinium-enhanced T1-weighted imaging with fat saturation [30].

Inflammatory MR lesions of the shoulders, the anterior chest wall and the pelvis have not been extensively investigated in AS [13,31]. There is some evidence, however, that inflammation of the hip joint may herald a poor prognosis [32,33].

Whole body MR imaging with the so-called total imaging matrix technology (TIM, Siemens Medical Solutions, Erlangen, Germany) reduces imaging times by virtually eliminating the need for patient repositioning and manual coil changes. Whole body MR imaging allows the visualization of inflammatory changes in the entire spine and the SI joints as well as of the shoulders, the anterior chest wall and the pelvis within 30 minutes including initial positioning of the patient. Spatial resolution is similar to standard MR examinations.

Inflammatory involvement of the lower thoracic spine was common both in the confirmed AS and suspected early AS group. This finding is in accordance with several studies using conventional MR imaging [13,17-19]. Inflammatory changes in the thoracic spine seem to be second in frequency only to inflammatory lesions in the SI joints. This finding may contribute to early diagnosis of AS and the lower thoracic spine should be included in MR imaging protocols analyzing active AS [17]. Inflammatory involvement of the lower lumbar spine without major changes in the SI joints has been shown occasionally [25,34].

In our study, inflammatory lesions of the SI joints were most frequently found in the inferior iliac quadrant in both groups. The remaining quadrants of the SI joints were more frequently affected in confirmed AS. An assessment of the SI joints by four quadrants seems to be justified by the consistent finding of early inflammation in the inferior iliac quadrant (dorsocaudal synovial part of the joint) [35,36]. A recently published study with MR-histological correlation of normal SI joints demonstrated that the characteristics of a synovial joint were confined to the distal cartilaginous portion at the iliac side [37].

Fatty replacement of subchondral bone marrow was common in our patients with confirmed AS. If this is a distinct feature of confirmed AS is still open for discussion [38]. Fat conversion of a mostly patchy distribution has been described in the sacral and iliac bone marrow in healthy volunteers without known inflammatory disease [15,39,40]. On the other hand, in a study of 41 patients with early spondyloarthritis, fat accumulation in the bone marrow was commonly associated with chronic inflammatory changes of the SI joints [12]. Based on the presence of joint fluid, the hip was relatively frequently involved in our study (N = 7). Additional bone marrow signal abnormalities of the left femoral head and acetabulum were detected in one patient in the confirmed AS group. This patient suffered from severe back pain with extensive inflammatory lesions of all segments of the spine but presented no signs of coxitis during the clinical examination. In two retrospective studies on the long term outcome covering a follow-up period of 38 and 10 years, respectively, hip joint involvement proved to be the single most important risk factor predicting ankylosis of the spine [32,33]. Whole body MR imaging is able to visualize inflammatory changes of the hip joints simultaneously with the SI joints and the spine.

Inflammatory changes in the manubriosternal joint were found in six patients, in the sternoclavicular joints in three patients and in the costosternal joints in one patient. Inflammatory involvement of the anterior chest wall in MR examinations has rarely been reported in AS [13].

Inflammatory lesions of the shoulders were confined to the confirmed AS group. The most frequently affected region was the acromioclavicular joint (N = 3). Inflammatory involvement with intense bone marrow abnormality at the supraspinatus insertion onto the greater tuberosity and acromial entheses of the deltoid muscle has previously been described as a common and specific finding in AS [31].

MR imaging is part of the Assessment in Ankylosing Spondylitis (ASAS) International Working Group recommendations for management of AS [41]. A collaborative initiative of ASAS and OMERACT (Outcome Measures in Rheumatoid Arthritis Clinical Trials) MR imaging in AS working group has been started with the goal to validate and standardize the available MR scoring systems for the spine and the SI joints, using standard MR systems [27]. The additional information on inflammatory involvement of the shoulders, of the anterior chest wall or of a possibly prognostically important coxitis may be of clinical importance. A systematic comparison of the quality of information gained by whole body MR imaging with the assessment of selected regions of the skeleton by conventional MR imaging techniques has not been performed in this study. However, the spatial resolution and other imaging parameters obtained with the MR system employed in this study are comparable to examinations dedicated to a specific body region.

## Conclusion

In conclusion, whole body MR examinations frequently demonstrate inflammatory lesions outside the SI joints in both confirmed AS and suspected early AS with a high disease activity judged on clinical grounds. These lesions are similarly distributed but occur less frequently in suspected early AS compared to confirmed AS. Due to the small sample size in this pilot study these results need to be confirmed in larger studies using this emerging technique.

Whole body MR imaging is a promising tool to assess the frequency and distribution of inflammatory lesions in the entire axial skeleton and the shoulder and pelvic girdle both in patients with suspected early and with confirmed active ankylosing spondylitis.

## List of abbreviations

AS Ankylosing Spondylitis

ASAS Assessment in Ankylosing Spondylitis International Working Group

BASDAI Bath Ankylosing Spondylitis Disease Activity Index

BASFI Bath Ankylosing Spondylitis Functional Index

BASMI Bath Ankylosing Spondylitis Metrology Index

CRP C-Reactive Protein

ESR Erythrocyte Sedimentation Rate

HLA B27 Human Leukocyte Antigen B27

MR imaging Magnetic resonance imaging

NSAID Non-Steroidal Antiinflammatory Drug

OMERACT Outcome MEasures in Rheumatoid Arthritis Clinical Trials

SAPHO Synovitis Acne Pustulosis Hyperostosis Osteitis syndrome

SI joints Sacroiliac joints

STIR Short Tau Inversion Recovery

TNFα-inhibitors Tumor Necrosis Factor alpha-inhibitors

## Competing interests

The author(s) declare that they have no competing interests.

## Authors' contributions

UW, RK, JH and MZ set up the study design. UW was responsible for the acquisition of the clinical data and drafted the manuscript. CP, MZ, JH and UW set up the MR imaging protocol. CP and MZ performed the readout of the MR examinations and were responsible for the descriptive statistics. RK, CP and MZ helped to draft the manuscript. All authors read and approved the final manuscript.

## Pre-publication history

The pre-publication history for this paper can be accessed here:



## Figures and Tables

**Figure 1:**
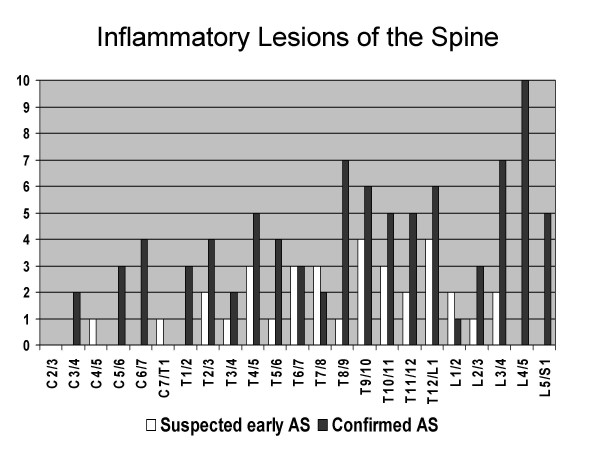
**Inflammatory lesions separated for each vertebral unit**. Numbers are patients with a positive finding. The white bars represent the patients with suspected early AS; the black bars represent the patients with confirmed AS.

**Figure 2:**
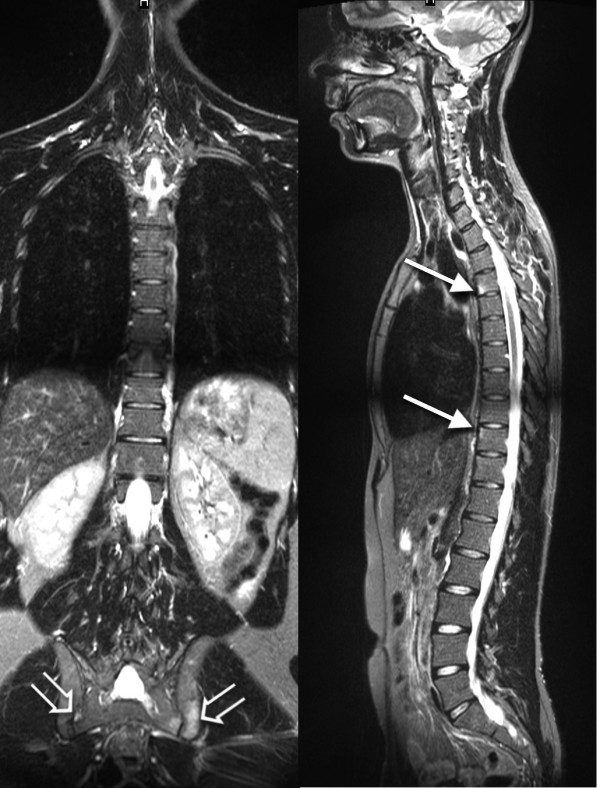
28-year old male patient with suspected early AS (duration of inflammatory back pain 7 months, BASDAI 3.7, BASDAI 2 5, HLA B27 positive). Coronal (left) and sagittal (right) STIR images show the most commonly seen signal abnormalities in suspected early AS. Inflammatory changes (open arrows) are seen in the lower aspects of the SI joints and subtle abnormalities in the anterior corner of the endplates in the thoracic spine (arrows).

**Figure 3:**
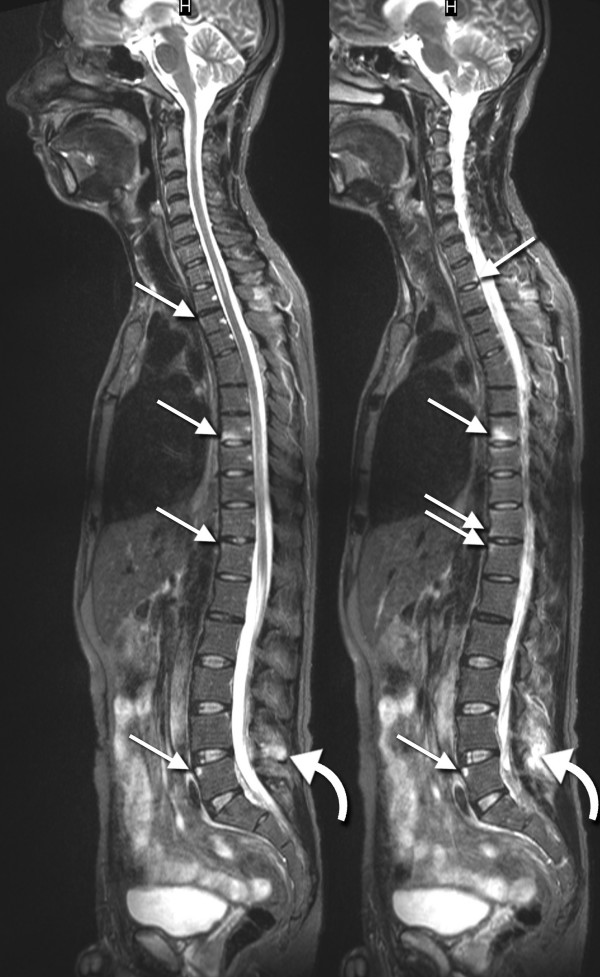
30-year old male patient with confirmed AS (duration of inflammatory back pain 7 years, BASDAI 4.8, HLA B27 positive). Sagittal STIR images show inflammatory lesions in the thoracic and lumbar spine (arrows). Inflammatory lesions of the spinous process are shown at L4 (curved arrow)

**Figure 5:**
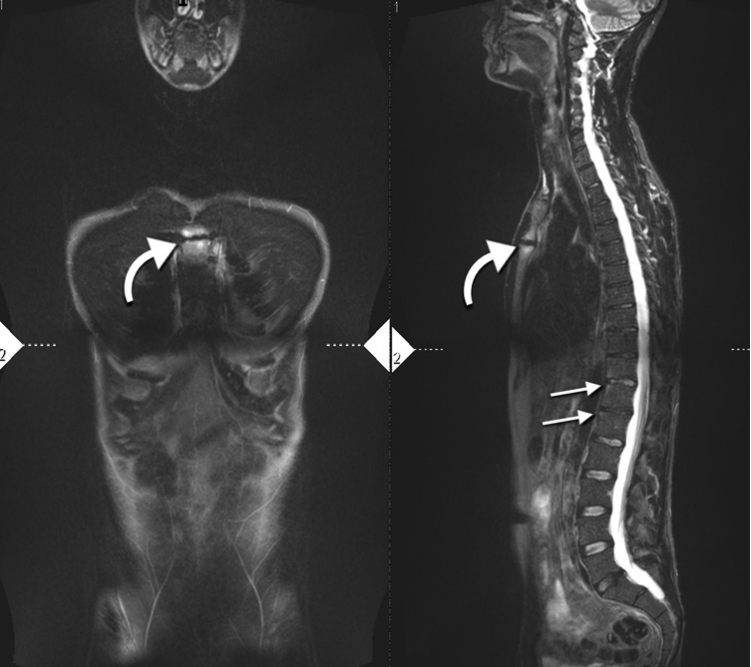
34-year old male patient with confirmed AS (duration of inflammatory back pain 13 years, BASDAI 4.9, HLA B27 positive). Inflammatory lesions of the anterior chest wall are shown in the manubriosternal joint (curved arrows) on coronal (left) and sagittal (right) STIR images. Inflammatory changes are seen in the lower thoracic spine and L1 (arrows).

**Figure 6:**
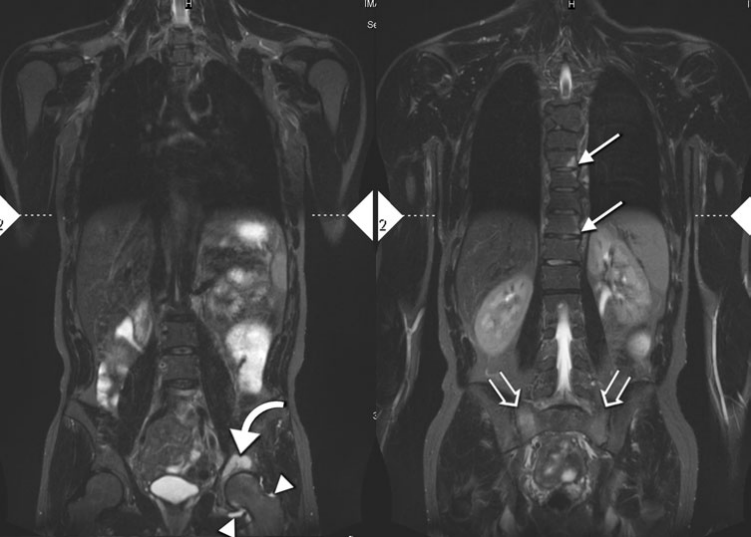
30-year old male patient with confirmed AS (duration of inflammatory back pain 7 years, BASDAI 4.8, HLA B27 positive). Two coronal STIR images demonstrate inflammatory lesions in the thoracic spine (solid arrows), in the sacroiliac joint (sacral side) (open arrows), and in the left hip. Extensive bone marrow changes in the acetabulum (curved arrow) and an effusion of the hip joint are shown (arrowheads).

**Figure 7:**
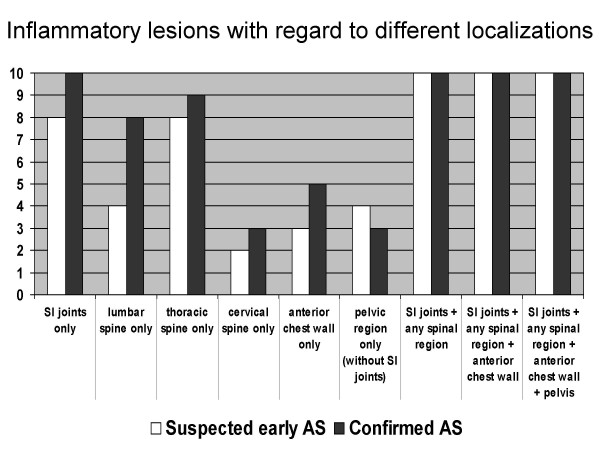
**MRI abnormalities suggestive of inflammatory lesions with regard to different localizations**. Numbers are patients with a positive finding. The white bars represent the patients with suspected early AS; the black bars represent the patients with confirmed AS. The wording "only" refers to inflammatory lesions when analyzing the selected region only irrespective of any additional inflammatory lesions seen in other localizations.

**Table 1:** Characteristics of the study patients

	Suspected early ankylosing spondylitis group (n = 10)	Confirmed ankylosing spondylitis group (n = 10)
Male : female	7 : 3	7 : 3
Age (years)	28.5 (21 – 45)	36.5 (30 – 48)
Disease duration (years)	0.8 (0.3 – 2.0)	12.5 (6 – 28)
HLA B27 positive (%)	90	90
BASDAI	4.6 (3.5 – 6.7)	5.9 (3.2 – 8.0)
BASDAI 2	6.0 (4 – 10)	7.0 (6 – 10)
BASFI	3.0 (0.5 – 6.2)	4.6 (0.8 – 7.8)
BASMI	1.0 (0 – 2)	2.0 (0 – 5)
ESR (mm/h)	8.0 (1 – 42)	16.5 (5 – 64)

Values presented are median (range) unless otherwise stated

BASDAI: Bath Ankylosing Spondylitis Disease Activity Index [21]; BASDAI 2: second question of the BASDAI instrument dealing with back pain; BASFI: Bath Ankylosing Spondylitis Functional Index [23]; BASMI: Bath Ankylosing Spondylitis Metrology Index [24]; ESR: Erythrocyte Sedimentation Rate

**Table 2:** Distribution of MR imaging lesions in the SI joints (four quadrants), in the spine (four regions), in the shoulder/pelvic girdle and the anterior chest wall

**SI Joints**: Inflammatory Lesions (four quadrants)				
	Suspected Early AS Patients		Confirmed AS Patients	

	Patients	Lesions	Patients	Lesions

Superior Ileal	2	2	5	8
Inferior Ileal	8	13	8	16
Superior Sacral	3	3	6	9
Inferior Sacral	6	8	10	15

**SI Joints**: Fatty Replacement of Bone Marrow (four quadrants)				

	Suspected Early AS Patients		Confirmed AS Patients	

	Patients	Lesions	Patients	Lesions

Superior Ileal	2	2	2	2
Inferior Ileal	3	4	6	10
Superior Sacral	3	3	6	9
Inferior Sacral	4	5	9	17

**Spine**: Inflammatory Lesions				

	Suspected Early AS Patients		Confirmed AS Patients	

	Patients	Lesions	Patients	Lesions

C-Spine (C2/3-C7/T1)	2	2	3	5
Upper T-Spine (T1/2-T6/7)	3	6	6	17
Lower T-Spine (T7/8-T12/L1)	7	15	9	21
L-Spine (L1/2-L5/S1)	4	5	8	18

**Shoulder/pelvic girdle and anterior chest wall**: Inflammatory Lesions				

	Suspected Early AS Patients		Confirmed AS Patients	

Sternoclavicular Joint	1		2	
Manubriosternal Joint	2		4	
Costosternal Junction	1		0	
Acromioclavicular Joint	0		3	
Glenohumeral Joint	0		0	
Subacromial Bursa	0		1	
Hip Joint	4		3	
Greater Trochanter	0		1	
Symphysis Pubis	0		1	
